# Brazilian guidelines on the frequency of ophthalmic assessment and recommended examinations in healthy children younger than 5 years

**DOI:** 10.5935/0004-2749.20210093

**Published:** 2025-08-21

**Authors:** Julia Dutra Rossetto, Luisa Moreira Hopker, Luis Eduardo M. Rebouças de Carvalho, Marcelo Gaal Vadas, Andrea Araújo Zin, Tomás Scalamandré Mendonça, Dirceu Solé, Luciana R. Silva, Christiane Rolim-de-Moura, Luis Carlos Ferreira Sá, Fabio Ejzenbaum

**Affiliations:** 1 Ophthalmology and Visual Science Department, Escola Paulista de Medicina Universidade Federal de São Paulo, São Paulo, SP, Brazil; 2 Pediatric Ophthalmology Department, Instituto de Puericultura e Pediatria Martagão Gesteira, Universidade Federal do Rio de Janeiro, Rio de Janeiro, RJ, Brazil; 3 Ophthalmology Department, Hospital de Olhos do Paraná, Curitiba, PR, Brazil; 4 Ophthalmology Department, Irmandade da Santa Casa de Misericórdia de São Paulo, São Paulo, SP, Brazil; 5 Instituto Nacional de Saúde da Mulher, da Criança e do Adolescente Fernandes Figueira, Fundação Oswaldo Cruz, Rio de Janeiro, RJ, Brazil; 6 Pediatric Department, Escola Paulista de Medicina, Universidade Federal de São Paulo, São Paulo, SP, Brazil; 7 Pediatric Department, Universidade Federal da Bahia, Salvador, BA, Brazil; 8 Ophthalmology Department, Instituto da Criança, Hospital das Clínicas, Faculdade de Medicina, Universidade de São Paulo, São Paulo, SP, Brazil

**Keywords:** Diagnostic techniques, ophthalmological, Vision screening, Vision tests, Child, Infant, Técnicas de diagnóstico oftalmológico, Triagem visual, Testes visuais, Criança, Lactente

## Abstract

**Purpose:**

To provide guidance on the frequency and components of eye examinations for healthy children aged 0 to 5 years.

**Methods:**

These guidelines were developed based on the medical literature and clinical experience of an expert committee. PubMed/Medline searches were performed, with selected publications not restricted to systematic reviews, randomized controlled trials, or observational studies. The Grading of Recommendations Assessment, Development, and Evaluation profile was applied when suitable, and for issues without scientific evidence, recommendations were based on expert consensus. Recommendations by the American Academy of Pediatrics, American Association of Pediatric Ophthalmology and Strabismus, American Academy of Ophthalmology, Royal College of Ophthalmologists, and Canadian Ophthalmological Society were also reviewed. The final guideline document was approved by the Brazilian Pediatric Ophthalmology Society and by the Brazilian Pediatric Society.

**Results:**

Newborns must undergo the red reflex test and inspection of the eyes and adnexa by a pediatrician within 72 hours of life. The red reflex test should be repeated by the pediatrician during childcare consultations at least three times per year during the first 3 years of life. If feasible, a comprehensive ophthalmologic examination may be performed between 6 and 12 months of age. Until 36 months of age, the pediatrician should assess the infant’s visual development milestones, age-appropriate assessment of visual function, ocular fixation, and eye alignment. At least one comprehensive ophthalmologic examination should be performed at 3 to 5 years of age. The examination should minimally include inspection of the eyes and adnexa, age-appropriate visual function assessment, evaluations of ocular motility and alignment (cover tests), cycloplegic refraction, and dilated fundus.

**Conclusions:**

Guidelines concerning the frequency of ophthalmic assessment are important tools for directing physicians regarding best practices that avoid treatable vision problems that affect children’s development, school, and social performance and cause unnecessary permanent vision loss.

## Abstract (pt)

**Objetivo:**

Fornecer orientações sobre a frequência e os componentes dos exames oftalmológicos para crianças saudáveis de 0 a 5 anos.

**Métodos:**

Essas diretrizes foram desenvolvidas com base em revisão bibliográfica e experiência clínica de um comitê de especialistas. Foram realizadas buscas PubMed/Medline; documentos selecionados não se restringiram a revisões sistemáticas, ensaios clínicos randomizados e estudos observacionais. Quando adequado, o perfil GRADE foi aplicado para graduá-los e o consenso de especialistas foi usado nos tópicos sem evidência científica. Também foram revisadas as recomendações pela Academia Americana de Pediatria, Associação Americana de Oftalmologia Pediátrica e Estrabismo, Academia Americana de Oftalmologia, *Royal College of Ophthalmologist* e Sociedade Canadense de Oftalmologia. O documento final foi aprovado pela Sociedade Brasileira de Oftalmologia Pediátrica e Sociedade Brasileira de Pediatria.

**Resultados:**

Os recém-nascidos devem ser submetidos ao teste do reflexo vermelho e inspeção dos olhos e anexos pelo pediatra dentro de 72 horas de vida ou antes da alta da maternidade. O teste do reflexo vermelho deve ser repetido pelo pediatra durante as consultas de puericultura pelo menos três vezes ao ano durante os primeiros 3 anos de vida. Se factível, um exame oftalmológico completo pode ser feito entre 6 a 12 meses de vida. Até os 36 meses de idade, os marcos visuais, função visual apropriada para a idade, fixação e alinhamento ocular também devem ser avaliados pelo pediatra ou médico da família. Pelo menos um exame oftalmológico completo deve ser realizados entre 3 e 5 anos de idade. O exame deve conter pelo menos inspeção dos olhos e anexos, avaliação da função visual apropriada para a idade, avaliação da motilidade e alinhamento ocular (testes de cobertura), refração sob cicloplegia e avaliação do fundo de olho dilatado.

**Conclusões:**

As diretrizes sobre a frequência da avaliação oftalmológica são ferramentas importantes para orientar os médicos sobre a melhor prática a fim de evitar problemas visuais tratáveis na infância, que poderiam comprometer seu desenvolvimento social, escolar e global, além de causar perda permanente da visão.

## INTRODUCTION

Guidelines are important tools that provide orientation to physicians regarding the best practices to achieve optimal health care. Well-established recommendations have been developed for visual screening during childhood in countries such as the United States, Canada, and the United Kingdom^(1-3)^. The lack of similar official guidelines in Brazil for the assessment of healthy children points to a need for local recommendations for childhood eye care that account for the regional specificities and characteristics of the Brazilian population and health system.

The objective of this document is to provide guidance on the frequency and components of eye examinations for healthy children aged 0 to 5 years. For this purpose, healthy children with age-appropriate neuropsychomotor development were considered.

## METHODS

These guidelines were focused on scientific rigor, clinical applicability, and editorial independence and sought clarity on communicating the recommendations. They were developed based on careful consideration of the medical literature and clinical experience of the expert committee of the Brazilian Pediatric Ophthalmology Society to define the optimal times and intervals for ocular assessments in healthy children until the age of 5 years and to establish which examinations should be recommended. For that purpose, searches of PubMed/Medline were performed in peer-reviewed journals written in Portuguese and English, using combinations of the following keywords: “vision screening,” “visual screening,” “ocular,” “ophthalmic,” “eye examination,” “vision test,” “diagnostic techniques, ophthalmological,” “vision disorders, diagnosis,” “eye diseases, diagnosis,” “visual acuity,” “refraction, ocular,” “amblyopia,” “refractive error,” “strabismus,” “child, preschool,” “infant,” and “pediatric”.

The documents considered were not restricted to systematic reviews, randomized controlled trials (no masked randomized clinical trial has evaluated the effectiveness of vision screening in children until 5 years of age), or observational studies. The Grading of Recommendations Assessment, Development and Evaluation (GRADE) profile was applied for the gradable articles; observational studies received the ranking of low grade of evidence^(4)^.

Because of the absence of strong scientific evidence in the literature, a 12-item questionnaire was designed to assess the recommendations of Brazilian ophthalmologists regarding a complete ophthalmologic examination before 1 year of life. The questionnaire was distributed to all members of the Brazilian Pediatric Ophthalmology Society (SBOP). Participants were characterized by their academic background and duration of practice in the field of pediatric ophthalmology. The percentage of pediatric patients in each participant’s regular practice, geographic location of care, and care peculiarities (primarily in public health versus private clinic) were also assessed. The main question was, “Do you recommend a complete ophthalmologic examination before 1 year of age in a healthy child?” A healthy child was defined as having no family history of eye disease, no systemic disease, no exposure to vertically transmissible diseases, born full-term, and with a normal red reflex test (RRT) performed in the maternity ward by the pediatrician.

For the descriptive analysis of the experts’ questionnaires, categorical variables were described as absolute and relative frequencies, whereas scores were presented as medians and percentile values (25th and 75th). Pearson’s chi-square test or Fisher’s exact test were used to determine statistically significant associations between the duration of clinical practice, scientific publication on pediatric ophthalmology, percentage of pediatric patients in regular practice, practice characteristics, and the recommendation regarding a comprehensive ophthalmologic examination before 1 year of age. The Mann-Whitney *U* test was also used to analyze differences between the percentage and total scores of academic backgrounds, duration of clinical practice in the area, and recommendation for a complete ophthalmologic examination before 1 year of age. The level of significance was 5%, and analyses were performed using SPSS, version 22.

Committee members also reviewed current recommendations by the American Academy of Pediatrics (AAP), the American Association of Pediatric Ophthalmology and Strabismus (AAPOS), the American Academy of Ophthalmology (AAO), the Royal College of Ophthalmologists (UK), and the Canadian Ophthalmological Society. The final guideline document was approved by the Brazilian Pediatric Ophthalmology Society, affiliated with the Brazilian Council of Ophthalmology, and by the Brazilian Pediatric Society.

Institutional review board approval was not obtained. This report does not contain any original data or personal information that could lead to the identification of patients.

## RESULTS

The questionnaire was answered by 193 members of the SBOP. Of these, 73.6% recommended a complete ophthalmologic examination in healthy children during the first year of life. There was a slight reduction in the percentage of recommendations associated with the increased duration of the ophthalmologist’s clinical practice (Table 1). With regard to the pediatric attendance percentage in regular practice, 82% of the professionals with percentages >75% recommended the examination, versus 64.7% of pediatricians who attend a minority of children (<25%), but this difference was not statistically significant. No association was noted between the domains “Academic background,” “Duration of clinical practice,” and “Scientific publication” and the recommendation for an examination in the first year of life. No association was observed between the total score composed by those domains and the recommendation for the examination.

**Table 1:** Analysis of the pediatric ophthalmologist questionnaire

	Recommendation of a comprehensive ophthalmologic examination at < 1 year of age		
	Yes	No	p-value
Duration of clinical practice			
Less than 5 years	33 (86.8)	5(13.2)	
Between 5 and 10 years	30 (75.0)	10(25.0)	
Between 10 and 20 years	39 (73.6)	14(26.4)	0.137
More than 20 years	40 (65.6)	21 (34.4)	
Scientific publication on pediatric ophthalmology			
No	99 (74.4)	34 (25.6)	
Yes	42 (71.2)	17(28.8)	0.724
Percentage of pediatric patient in regular practice			
Less than 25%	22 (64.7)	12 (35.3)	
Between 25 and 50%	31 (64.6)	17(35.4)	
Between 50 and 75%	39 (78.0)	11 (22.0)	0.109
More than 75%	50 (82.0)	11 (18.0)	
Practice characteristics			
Public health (>75%)	13 (76.5)	4 (23.5)	
Public health and private practice (50%-50%)	43 (76.8)	13 (23.2)	0.794
Private practice (>75%)	86 (71.7)	34 (28.3)	
Score			
Academic background (0-3)	1 [1-2]	1 [0-2]	0.217
Duration of clpnical Practice (0-2)	1 [0-2]	1 [0-2]	0.063
Scientific publications (0-5)	0 [0-1]	0 [0-2]	0.484
Total (0-10)	2 [1-4]	2 [1-4]	0.669
Score (%)	20 [10-40]	20 [10-40]	0.669

* Values expressed as n (%) or median [25th percentile-75th percentile].

## RECOMMENDATIONS (BOX 1)

A child with age-appropriate neuropsychomotor development should be considered healthy, especially in the absence of the following:

**Box 1:** Recommendations of the Brazilian Pediatric Ophthalmology Society

Brazilian Pediatric Ophthalmology Society Recommendations
- Red reflex test (RRT) should be performed within 72 hours of life and repeated
by the pediatrician during childcare consultations at least three times a year
during the first 3 years of life.^*^
- Routine age-appropriate visual function assessment of infant and child during
the first 3 years of life should be performed by a primary health care provider
or pediatrician.^*^
- Children aged 6 to 12 months may undergo a comprehensive ophthalmological
examination (2C)
- Children aged 3 to 5 years (ideally by age 3 years) should undergo a comprehensive
ophthalmological examination (1B).

* Failure of visualization of RRT or the presence of any eye abnormalities are indications for a referral to an ophthalmologist.

1. apparent eye abnormality (e.g., leukocoria, ptosis, nystagmus, or strabismus),
2. extreme prematurity (babies ≤1500 g or ≤32 weeks of gestational age),
3. exposure to vertically transmissible infectious diseases (such as toxoplasmosis, syphilis, cytomegalovirus, or Zika virus),
4. diseases associated with ocular manifestations (e.g., metabolic disorders, juvenile idiopathic arthritis, or Down syndrome),
5. family history of eye diseases in childhood (such as cataracts, glaucoma, or retinoblastoma), and
6. clinical suspicion of a visual deficit.

In the presence of any of the above, a comprehensive eye examination by an ophthalmologist, ideally within 1 month of the recognition of the affliction, is recommended, regardless of the result of the RRT^(5,6)^.

**Newborns**: The RRT must be performed by the pediatrician within 72 hours of life or before discharge from the maternity ward.

NOTE: The RRT should be repeated by the pediatrician during childcare consultations at least three times per year during the first 3 years of life^(7)^. Failure of visualization or abnormalities of the reflex are indications for an urgent referral to an ophthalmologist.

**0 to 36 months**: Inspection of the eyes and adnexa (lids, cornea, conjunctiva, iris, and pupil), age-appropriate assessment of visual function, ocular fixation, and eye alignment should be performed by a primary health care provider or pediatrician^(3,7,8)^.

**0 to 12 months:** Assess visual development milestones for healthy infants:

- 1 month old: visual fixation present;
- 2 months old: develops vertical eye movements;
- 3 months old: follows objects; demonstrates adequate saccadic movements (note that eye movements may not be coordinated until 6 months of age);
- 6 months old: reaches for objects, has appropriate eye alignment; and
- 9 months old: recognizes faces and expressions.
- NOTE: Infants who do not make eye contact in the first 2 months of life or do not show social smiling or perception of their own hands at 3 months, who do not pick up toys at 6 months, or who do not recognize faces at 11 months should be considered for a comprehensive eye examination^(3)^.

**12 to 36 months**: Assess the following in both eyes, each eye separately:

- fixation (if central, steady, and maintained),
- the ability to follow light and objects; and
- the reaction to the occlusion of each eye.

**Between 6 and 12 months:** A comprehensive eye examination could be performed by an ophthalmologist, including inspection of the eyes and adnexa, visual function assessment (monocular fix-follow examination), evaluation of ocular motility and alignment (simple and alternate cover tests), and cycloplegic refraction and dilated fundus evaluation (Recommendation 2C)^(9,10)^.

**Between 3 and 5 years (ideally at 3 years)**^(11-14)^: A comprehensive eye examination should be performed by an ophthalmologist, including inspection of the eyes and adnexa, evaluation of visual acuity (with age-appropriate optotypes), evaluation of ocular motility and alignment (simple and alternate cover tests), and cycloplegic refraction and dilated fundus evaluation (Recommendation 1B)^(6,9)^.

If the examination is inconclusive or unsatisfactory, a new assessment is recommended within 6 months. Between 5 and 8 years of age, children should ideally be submitted to annual monocular vision screening, and those who fail (visual acuity of less than 20/40 in at least one eye) should undergo a comprehensive eye examination.

## DISCUSSION

Uncorrected refractive errors can be responsible for up to 69% of the visual problems that occur in childhood^(15)^. Depending on the degree of refractive error and the child’s age, these problems can be potentially amblyogenic if not corrected; that is, they can lead to visual impairment^(16)^. In school-age children, uncorrected refractive errors are considered a public health problem and are the main cause of visual impairment in children around the world. According to the World Health Organization and the Brazilian Council of Ophthalmology, an estimated 23 million children in Latin America have vision problems related to uncorrected refractive errors that can affect their development, schooling, and social performance^(17)^.

If detected and treated early, amblyopia is the second most treatable eye disease (after refractive errors)^(11-14,18)^, and it affects about 2%-4% of the pediatric population^(16,19)^. It is usually treatable if diagnosed early; however, because of the difficulty in detecting the disease, hundreds of thousands of children in the United States and millions around the world suffer unnecessary and permanent vision loss each year^(7,16)^. In general, the benefits of screening and treating amblyopia outweigh the costs and any possible damage^(20-22)^. Untreated amblyopia can have a negative effect on visual function, quality of life, and labor capacity^(19)^. For this reason, the treatment of amblyopia has been proven to be one of the most cost effective medical procedures in the world^(23,24)^. The presence of strabismus can lead to the suppression of the nondominant eye and may ultimately be the cause of amblyopia in up to 50% of patients^(25)^.

Uncorrected refractive errors are the most common finding in ophthalmological examinations and are the easiest to correct; however, they are not easily identified by a simple examination of visual acuity and ocular motility^(26-28)^. Amblyopia may be suspected after a basic visual screening, but treatment requires a complete eye evaluation^(11,20)^. Examination techniques for amblyopia detection and treatment in children younger than 5 years may include the assessment of monocular fixation and visual acuity, red reflex and corneal reflex tests, sensory fusion, stereopsis, biomicroscopy, simple and alternate cover tests, ocular motility, and cycloplegic refraction and dilated fundus evaluation. Complementary examinations include tests of pupillary reflex and color vision^(6,9,16)^.

Visual screening is the most efficient approach for early detection of ocular disease. Screening should be initiated by the pediatrician in newborns through the RRT before discharge from the maternity ward and in subsequent childcare consultations^(5,7,8,16,19)^. The pediatrician should also assess visual development milestones as part of the sensory-motor and growth milestones followed through childcare.

A report on visual screening in childhood, drawn up in consensus between the AAP, AAPOS, and AAO, recommends that newborns be examined using the RRT for eye abnormalities such as cataracts, corneal opacity, and ptosis^(6,8)^. The AAP also recommends that all children up to 6 months of life undergo examinations for ocular fixation, eye alignment, and the presence of ophthalmic disease. The Child Eye Care Guidelines and the AAO indicate that periodic assessments of visual function can be performed by an ophthalmologist, pediatrician, or trained health professional. Children referred to an ophthalmologist should be seen as soon as possible, ideally within 3 months^(3)^.

The present guideline recommends that the pediatrician or family physician perform the periodic assessment of RRT (at least three times per year during the first 3 years of life), the inspection of the eyes and adnexa (lids, cornea, conjunctiva, iris, and pupil), and the age-appropriate assessment of visual function, ocular fixation, and eye alignment from birth until 36 months of life^(3,7,8)^. A complete ophthalmological examination between 6 and 12 months can be performed, especially in locations that have sufficient professionals for this purpose and/ or where visual evaluation by a pediatrician or family physician is unavailable. This recommendation was also made by most pediatric ophthalmologists in Brazil (76.3%), regardless of their geographic location, academic background, duration of clinical practice, or site of practice (public or private health system).

To date, no masked randomized clinical trial has evaluated the effectiveness of vision screening in children aged 0 to 5 years. However, prospective cohort studies have provided evidence that multiple visual screenings in the first 6 years of life reduce the prevalence of amblyopia at 7 to 8 years of age^(11-14,21)^. Identifying the best age for screening in children based on evidence is more challenging^(2)^. The US Task Force Recommendation Statement advises single visual screening between the ages of 3 to 5 years and refers to insufficient evidence for screening children younger than 3 years^(29)^. One community-based program with a significant sample (more than 200,000 tests) demonstrated that photoscreening in children younger than 3 years and between 3 and 5 years of age has the same ability to detect amblyogenic risk factors and therefore recommends early screening in children 1 year of age or older, before amblyopia is more pronounced^(30)^. Groenewoud et al. suggested that preschool screening from the age of 3 years is superior for the detection of amblyopia^(21)^. Donahue et al. studied children with anisometropia referred by a photoscreening program and reported that the prevalence of amblyopia increased proportionally with age but remained relatively constant after 4 years, and that the severity of amblyopia also increased with age^(31)^. In summary, they reported that children with anisometropia are less likely to have amblyopia if their condition is detected at an early age and that amblyopia is generally established by 3 years of age^(31)^.

The previously reported joint policy statement recommends that instrument-based screening, when available, should be attempted by the age of 2 years and that direct testing of visual acuity should begin by age 3, using age-appropriate optotypes^(3,6)^. Repeated screenings are important in childhood, as a single assessment may be insufficient for different reasons, depending on the child’s age group and cooperation. If the child is unable to undergo the screening test, the test must be repeated within 6 months, because repetition of the examination increases the probability of detecting a visual problem. Following these data, we recommend at least one ophthalmological comprehensive examination between 3 and 5 years of life and ideally at 3 years of age because of the better prognosis for early treatment of amblyopia.

In Brazil, some states have already passed legislation that makes it obligatory for the pediatrician to perform the RRT in all newborns before discharge from the maternity ward. The Supplementary Health National Agency has also included the RRT in the list of procedures with mandatory coverage by health insurance^(17)^. If the RRT result is abnormal, the child should be referred immediately to an ophthalmologist, because congenital cataract is a possible cause and may require surgery before 12 weeks of life^(7,16,19)^. Conversely, reliable data are lacking regarding the prevalence and causes of blindness among children in Brazil^(17)^ and no comprehensive social programs are available for periodic visual screening, and no non-ophthalmologist professionals are qualified to perform these examinations nationally. In addition, eye fixation and alignment tests are generally not performed by pediatricians or family doctors. Therefore, assessing visual acuity and performing eye examinations for the early diagnosis of diseases are ultimately the responsibility of ophthalmologists who have experience treating children. This is one of the main reasons why most Brazilian pediatric ophthalmologists recommend early examinations. Following the recommendations for eye examinations practiced in countries such as the United States, in which regular visual screening is practiced throughout childhood, is also unfeasible in Brazil.

Taking into consideration all of the information discussed in this document, the SBOP proposes that, in addition to ocular monitoring by the pediatrician or primary health care provider, at least one comprehensive ophthalmological examination should be performed between the ages of 3 and 5 years in all healthy children. If feasible, this could be preceded by an examination at 6 to 12 months of age. Regardless of the recommendation of the later examination by specialists, the context of the Brazilian Unified Health System must be taken into account, because the huge shortage of professionals presents a difficulty in delivering even a single examination before the age of 5 years. In the city of Rio de Janeiro alone, 4505 children are currently awaiting an ophthalmological appointment^(32)^.

It is ideal for children between the ages of 5 and 8 years to undergo an annual vision screening performed by non-ophthalmologists, with patients who fail (i.e., visual acuity of less than 20/40 in one eye) referred for an ophthalmological examination. Hence, public health policies must be designed to carry out this evaluation, which could, for example, be performed in schools. Another action could be the incorporation of visual acuity measurement into the pediatrician’s practice, but this would demand a readjustment in their offices, routine visits, and codes covered by health insurance. In the absence of universal pediatric screening by non-ophthalmologists, and where ophthalmologists are available, an annual examination with the latter is an alternative suggested by the SBOP until an effective screening protocol is established.

Guidelines are flexible tools that are based on the best scientific evidence and the clinical information available. They also reflect the consensus of experts in the field and allow them to use their judgment in the management of their patients^(33)^. Guidelines are not intended to provide step-by-step medical care or to replace clinical judgment; on the contrary, their intention is to support standards of practice.^^2^^ This guideline written by the SBOP should be considered in this context. Adhering to its recommendations will not necessarily produce successful results in all cases.

This guideline is also not intended to define or serve as a legal standard for medical care; therefore, it should not be used as a legal resource, because its general nature cannot provide individualized guidance for all patients in all circumstances^(2,34)^. Our target audience is ophthalmologists and pediatricians who evaluate Brazilian infants and children. The recommended examination intervals may also be of interest to the general public and public health policy makers. Given the diversity in the financial and health structure of different regions in Brazil, this guideline could serve as a basis for the defense of basic eye care for the pediatric population in needy areas.
